# Personal

**Published:** 1902-09

**Authors:** 


### PERSONAL.

Dr. Jane W. Carroll, of Buffalo, was recently appointed supreme medical examiner of the Ladies' Catholic Benevolent Association. At a reception lately given to Dr. Carroll by the local branch of the association, the president, Mrs. Mary Lee, congratulated Dr. Carroll on the honor which had been conferred upon her. This association of women comprises 80,000 members, and in the name of the Buffalo branch Mrs. Lee presented her a beautiful floral gift. Dr. Carroll made fitting response.

---

Dr. F. W. Jelks, who has been engaged in practice in St. Louis for some years, has removed to Hot Springs, Arkansas, and will take charge of the practice of his father, the late Dr. James T. Jelks. He will also assume charge of the *Hot Springs Medical Journal*, and will become one of the associate physicians of the Ozark Sanatorium.

---

Dr Matthew D. Mann, of Buffalo, sailed for Europe, August 6, 1902, for a short vacation. During his absence he will attend the International Congress of Obstetrics and Gynecology, at

Rome. Dr. Mann has been chosen an honorary president for America at this congress. He is accompanied by Mrs. Mann.

Dr. Harvey R. Gaylord, of Buffalo, departed for Europe about the middle of August, to remain for an extended period in pursuit of his studies at the German universities. He was accompanied by Mrs. Gaylord.

Dr. John H. Grant, of Buffalo, has removed his residence from Hamburg to the Algonquin, 76 Johnson Park, and his office from 893 Ellicott Square to 715 Mutual Life Building.

